# Influence of the mutation load on the genomic composition of hybrids between outcrossing and self‐fertilizing species

**DOI:** 10.1002/ece3.10538

**Published:** 2023-09-15

**Authors:** Fréderic Fyon, Waldir M. Berbel‐Filho

**Affiliations:** ^1^ Department of Biology Royal Holloway University of London Egham UK; ^2^ Department of Biology University of Oklahoma Norman Oklahoma USA

**Keywords:** hybridization, mating systems, mutation rate, mutational load, recombination, self‐fertilization

## Abstract

Hybridization is a natural process whereby two diverging evolutionary lineages reproduce and create offspring of mixed ancestry. Differences in mating systems (e.g., self‐fertilization and outcrossing) are expected to affect the direction and extent of hybridization and introgression in hybrid zones. Among other factors, selfers and outcrossers are expected to differ in their mutation loads. This has been studied both theoretically and empirically; however, conflicting predictions have been made on the effects mutation loads of parental species with different mating systems can have on the genomic composition of hybrids. Here, we develop a multi‐locus, selective model to study how the different mutation load built up in selfers and outcrossers as a result of selective interference and homozygosity impact the long‐term genetic composition of hybrid populations. Notably, our results emphasize that genes from the parental population with lesser mutation load get rapidly overrepresented in hybrid genomes, regardless of the hybrids own mating system. When recombination tends to be more important than mutation, outcrossers' genomes tend to be of higher quality and prevail. When recombination rates are low, however, selfers' genomes may reach higher quality than outcrossers' genomes and prevail in the hybrids. Taken together, these results provide concrete insights into one of the multiple factors influencing hybrid genome ancestry and introgression patterns in hybrid zones containing species with different mating systems.

## Graphical abstract

Genomic composition of hybrids between species with different mating systems (selfers and outcrossers) can be influenced by many parameters, including recombination rates, mutation rates, and hybrid mating systems. Here, we show that genes from the parental species with least mutation load tend to prevail, and that hybrid's own mating system appears to have only a limited influence on hybrid ancestry.

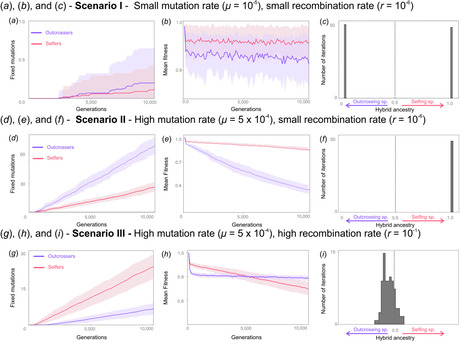

## 1 INTRODUCTION

Hybrid individuals are defined as progeny of mixed ancestry; they bear genes inherited from divergent evolutionary lineages. Introgressive hybridization (i.e., formation of hybrids with backcrossing) functions as bridges between divergent lineages, allowing gene flow between diverging lineages. Identifying the processes shaping the evolutionary fate of hybrid populations is a fundamental task for evolutionary biology, conservation, and wildlife management.

The possibility of hybridization happening, along with its ecological and genomic consequences, depends, among other factors, on the mating systems and reproductive biology of the parental species. Most plants and many animals are simultaneous hermaphrodites (Jarne & Auld, 2006; The Tree of Sex Consortium, 2014). In these species, self‐fertilization (selfing)—offspring produced by the fusion of gametes stemming from different meiosis in the same individual—is a possibility. Selfing contrasts with outcrossing as in the latter, the progeny is produced by the fusion of gametes proceeding from different meiosis in different individuals (hermaphrodites or not). Mating systems are usually defined in three main categories according to the selfing rate: obligate outcrossing (selfing rate ≤ 20%), predominantly selfing (selfing rate > 80%), and mixed‐mating (selfing rate > 20% and ≤ 80%) (Shimizu & Tsuchimatsu, 2015). It is only recently that attention has been brought on the effects that different mating systems have on the occurrence of hybridization and on the genomic composition of hybrid populations (Hu, 2015; Kim et al., 2018; Pickup et al., 2019).

Hybrid zones are regions of contact between divergent species/lineages that often produce individuals of admixed ancestry through interspecific mating. Hybrid zones between species with different mating systems (e.g., predominantly selfing and obligate outcrossing) are commonly reported in plants (Ostevik et al., 2021; Pickup et al., 2019; Ruhsam et al., 2011) but examples are scarce in animals (Berbel‐Filho et al., 2021). Those hybrid zones are often possible because of extensive variation in selfing rates between populations of a same species (Jarne & Auld, 2006; Whitehead et al., 2018), providing opportunities for hybridization between lineages with different mating systems. Some predictions on how reproductive systems (Brandvain & Haig, 2005) and reproductive timing (Berbel‐Filho et al., 2021; Martin & Willis, 2007), along with other ecological factors (Busch et al., 2022), can influence the occurrence and outcome of hybridization in these cases have been extensively provided. On the contrary, few studies have tried to address how differences in parental species' mating systems, which often translate into inter‐specific genetic variation, influence how the genetic composition of hybrids evolves in the long term (Hu, 2015; Kim et al., 2018; Pickup et al., 2019). Disparate, sometimes contradictory predictions on this have been made; the present work was designed to disentangle these and provide a unified framework.

Predictions concerning hybrids' genetic composition and evolution have focused on mutation load. They rely on the idea that species with different mating systems are expected to harbor different mutation loads (Arunkumar et al., 2015; Pickup et al., 2019). High homozygosity caused by long‐term selfing means that recombination is—most of the time—inefficient at shuffling genetic combinations. Therefore, selfers are expected to suffer from particularly strong selective interference—the process by which selection ongoing on one locus produces indirect frequency changes at linked loci (Wright et al., 2013). Selection is thus expected to be less efficient in selfing populations: they are predicted to have more slightly deleterious alleles become fixed by random genetic drift (Charlesworth et al., 1993; Hartfield et al., 2017; Hartfield & Glémin, 2014, 2016). This relates to the classic argument that the absence of effective recombination in asexual and self‐fertilizing species should lead them to extinction due to mutational meltdown (Abu Awad & Billiard, 2017; Gabriel et al., 1993; Lynch et al., 1993, 1995; Willi, 2013) (though this theoretical prediction has received mixed empirical support, see (Escobar et al., 2010) for example). Altogether, this predicts that genomes of self‐fertilizing species tend to be of worse quality. On the other hand, highly deleterious recessive alleles are expected to be purged by selection in selfers as they are “exposed” to selection by high homozygosity, while in outcrossers they can be maintained at low frequencies as they are partly hidden from selection in heterozygotes (Wang et al., 1999). In other words, here selection is predicted to be more efficient in self‐fertilizing species, resulting in genomes of better quality in selfers (Abu Awad & Roze, 2018). Overall, selfers' genomes are expected to harbor more fixed slightly deleterious, codominant mutations, while outcrossers' genomes are predicted to host more fixed strongly deleterious, recessive mutations (Arunkumar et al., 2015). How are these two alternative mutation loads expected to interact and determine the genetic composition and evolution of hybrids between selfers and outcrossers?

The answer to this question is likely to depend on several parameters of the selection processes in parental species but also on the hybrid mating system. Highly deleterious, recessive mutations coming from outcrossing ancestors may rapidly be purged by selection if the hybrids self‐fertilize (as these mutations rapidly become homozygous and visible to selection), taking together any linked genes. This process is likely to limit the introgression of outcrossers' genomes into selfers' (Pickup et al., 2019). On the other hand, the many fixed slightly deleterious, codominant mutations coming from the selfing ancestors should be purged out in an outcrossing hybrid, this time restricting introgression of self‐fertilizers' genomes into outcrossers' (Pickup et al., 2019). Under this argument, everything happens as if each parental genome was “adapted” to the mating system it has evolved in and tends to fare worse in alternative mating systems. This raises the question, does the mating system of the hybrid population have a critical influence on the evolution of hybrid genome composition, or is it simply determined by the respective quality of the parental populations' genomes?

Here, we aim to answering these questions by building a multi‐locus model allowing for selection, recombination, mutation, and drift to interplay and modify diploid genomes. Running numerical simulations for a diversity of scenarios, we were able to study the effects of selective interference and homozygosity on (1) the relative mutation loads of self‐fertilizing and outcrossing populations and (2) the long‐term genetic composition of hybrid populations deriving from parental populations with different mating systems.

First, our work allows us to confirm some of the classic arguments on mutation load in self‐fertilizing species. As another theoretical study showed recently (Sianta et al., 2022), how mutation loads in self‐fertilizing and outcrossing populations compare with one another strongly depends on the recombination rate between selected loci. At high recombination rates, outcrossers appear to be more efficient at limiting mutation load. However, at low recombination rates, outcrossers suffer from selective interference just as much as selfers; the latter better purge strongly deleterious recessive mutations, resulting in them having a lesser mutation load relative to outcrossers.

Second, we were able to determine the parameters that have a notable influence on the genetic composition and evolution of the hybrid population. Interestingly, we show that the mating system of the hybrid population does not largely influence the outcome of the simulations. Hybrid genetic composition in the long term appears to be primarily dictated by the relative mutation load of the parental species: genomes of worse quality get preferentially eliminated. Genomic mutation rate, recombination rate, as well as the reproductive mode of the parental species all influence how differently the mutation load builds up in the parentals, and thus how hybrid genome composition evolves in the long term. These results provide a new key to understand empirical data related to hybrid genome composition and patterns of introgression in hybrid zones between selfer and outcrosser species. Depending on genomic (recombination rates, mutation rates), ecological (intensity of selection), and demographical (population size, bottlenecks, colonization events) parameters, one can now predict how mutation load may have built in the parental species, and how much this could have contributed to hybrid genome composition.

## 2 METHODS

We used SLiM3 (Haller & Messer, 2019) software to code and run the model. SLiM3 has been used in a diversity of population genetics studies as it provides a resource‐effective and flexible computing framework. Because the model is of a stochastic nature, each simulation is repeated 100 times, and mean values as well as standard deviations are calculated from these 100 iterations. The code used here is available on the following GitHub repository: https://github.com/FredericFyon/Mating‐systems.

### 2.1 Theoretical populations

Our aim here is to follow the genetic alleles at different loci in populations with different mating systems, along with their hybrids. To do so, we always model three populations: (1) an exclusively or mostly self‐incompatible (SI) population that reproduces via obligate outcrossing (outcrossers); (2) a self‐compatible (SC) population that reproduces exclusively or mostly by means of self‐fertilization (selfers); (3) a population born by hybridization of the two previous ones. Populations do not interact with each other (except at the one generation where hybridization happens) and do not compete. The three populations, including the hybrid one, all have the same demographic size *N*_pop_. In the following, we call *σ*_*i*_ the rate of self‐fertilization of a given population *i*. We refer to the self‐fertilization rate of the outcrossing, self‐fertilizing, and hybrid populations as *σ*_o_ ≤ 0.1, *σ*_s_ ≥ 0.9 and *σ*_h_, respectively.

### 2.2 Genetic architecture

The genome of all individuals in the three populations consists of *N*_loc_ loci, equally distributed along a chromosome. We note *r* the recombination rate between two adjacent loci. We consider here an infinite‐allele model: each mutation occurring is unique, all loci potentially have an infinite number of alleles. In this model, we only consider deleterious mutations and note *s* the detrimental effect of a particular mutation on the fitness of the host individual.

### 2.3 Life cycle

At each generation, selection is modeled by choosing the parents of every offspring born in this generation. To do that, we first determine if an offspring was produced by self‐fertilization or not. With probability *σ*, it is produced by self‐fertilization. In that case, we randomly draw one parent from the distribution of individuals of the same population at the previous generation. In contrast, with probability 1–*σ*, the offspring is producing by outcrossing: we randomly draw two parents. Drawn parents are then accepted with a probability equal to their fitness *w*_*i*_. We assume fitness to be purely multiplicative among loci (we assume no epistasis): $w_{i}=\prod_{j}w_{\mathrm{ij}}$, with *w*_*i,j*_ being the contribution of locus $j\in \left[1,N_{\mathrm{loc}}\right]$ to the fitness of individual *i*. *w*_*i,j*_ depends on (1) the detrimental effect $s_{\mathrm{ij}}^{1}$ and $s_{\mathrm{ij}}^{2}$ of the alleles present on the two homologous chromosomes at locus *j* of individual *i*, and on (2) the dominance coefficients $h_{\mathrm{ij}}^{1}$ and $h_{\mathrm{ij}}^{2}$ of these two alleles. Following standard population genetics design, if the alleles are not identical‐by‐descent (that is, if the individual is heterozygote), *w*_*i,j*_ is calculated as:

$$w_{i,j}=\prod_{k=1}^{2}\left(1-h_{\mathrm{ij}}^{k}s_{\mathrm{ij}}^{k}\right)$$

If they are identical‐by‐descent (homozygote), then *w*_*i,j*_ simply is: $w_{i,j}=1-s_{\mathrm{ij}}$ (with $s_{\mathrm{ij}}^{1}=s_{\mathrm{ij}}^{2}=s_{\mathrm{ij}}$).

Following empirical data suggesting that the dominance of a deleterious mutation is negatively correlated with its detrimental effect (Agrawal & Whitlock, 2011; Phadnis & Fry, 2005; Simmons & Crow, 1977), we assume in this model a negative exponential relationship between *s* and *h*:

$$h_{\mathrm{ij}}^{k}=\frac{e^{-\alpha s_{\mathrm{ij}}^{k}}}{2}$$

with α being an arbitrarily chosen parameter that allows to modulate this relationship. Here, we will always assume that α=7 for simplicity. This allows to approximately match empirical data of mean dominance of mildly deleterious mutations (*s =* 0.1) being around 0.25 (Manna et al., 2011; however, see García‐Dorado & Caballero, 2000; Simmons & Crow, 1977; for example, for other estimates). The exponential function allows for a long‐tailed asymptotic distribution of dominance effects, letting highly deleterious mutations to have a very low but strictly positive dominance. Also, the function means that slightly deleterious mutations approach codominance. This allows us to incorporate in the model deleterious mutations in the whole range of dominance values from almost complete recessivity to almost codominance. Overall, this allows the whole selective process to be determined by only one parameter: the distribution of detrimental effects of deleterious mutations.

In addition to selection, the life cycle includes recombination and mutation. Recombination happens at a rate *r* being any two adjacent loci. A recombination event exchanges the composition of the two homologous chromosomes in an individual from the recombination point to the downstream end of the chromosomes. Of course, multiple recombination events can take place during any meiotic event, leading to potentially mosaic‐inherited chromosomes.

Mutations happen with probability *μ* per locus per chromosome per individual. When a mutation occurs, we draw its detrimental effect from a negative exponential distribution of mean 1/*λ*. This means that there is a vast majority of slightly deleterious, codominant mutations arising, along with some mildly deleterious, recessive mutations and very few highly deleterious, strongly recessive mutations.

### 2.4 Hybridization

At first, we consider only the parental populations of outcrossers and selfers. These reproduce within themselves for a certain number of generations *t*_1_. In our simulations, we mostly used *t*_1_ = 10,000 (except in Figure 4 where we illustrate the effect of varying *t*_1_). Then, a hybridization event happens. Hybridization is modeled by creating a third, hybrid population. This population is created by drawing for each individual one parent from each parental population. As a result, at *t*_1_ + 1, every individual of the hybrid population bears one chromosome from one parental population, and the other from the other parental population. After that initial hybridization event, we consider that there is no additional gene flow from the parental populations (as backcrossing would blur the sole effect of mutation load). We let the hybrid population reproduce within itself and evolve in the absence of additional mutation during 20,000 generations. This allows us to determine the evolutionary fate of the parental genomes within the hybrid population.

### 2.5 Parameter values

Unless specified otherwise, simulations throughout this work have been run with the following parameter values: *N*_pop_ = 500, *N*_loc_ = 100, λ = 10, *σ*_o_ = 0 (purely outcrossing population), and *σ*_s_ = 1 (purely self‐fertilizing population). We discuss later the influence of these parameters and provide examples with some other values in the Appendix S1.

## 3 RESULTS

### 3.1 Mutation load in outcrossers and selfers

We present in the Appendix S1 some results on how mutation load builds up in outcrossing and self‐fertilizing populations. We only rapidly present these here as they are already known patterns (Sianta et al., 2022).

Following classic population genetics arguments, our model shows that outcrossers' genomes present higher heterozygosity, more deleterious mutations, and lower dominance coefficients than selfers' genomes (see Figure S1).

Our results also emphasize that the relative rates of fixation of deleterious mutations in outcrossers and selfers highly depend on mutation and recombination rates. Higher mutation rates mean more fixations (Figure S2a,c,e). Interestingly, we see that when the recombination rate is large, selfers accumulate more fixed deleterious mutations (Figure S2b,d,f) as they suffer from selective interference: high rates of homozygosity (Figure S1e) mean recombination is mostly inefficient at reshuffling genetic associations. However, when recombination rates are small, outcrossers suffer from selective interference just as much as selfers. Consequently, outcrossers may accumulate more deleterious mutations than selfers (Figure S2c,e), as they suffer from a reduction of selection efficacy associated with the detrimental effect of mutations being partially hidden in heterozygotes.

Overall, this allows us to determine areas of the parameter space where outcrossers' genomes are of better quality (high recombination rates) and areas of the parameter space where selfers' genomes are of better quality (low recombination rates). Parting from here, we can investigate how these genomes fare when put together in hybrids.

### 3.2 Genetic composition of hybrids between Outcrossers and Selfers

**FIGURE 1:**
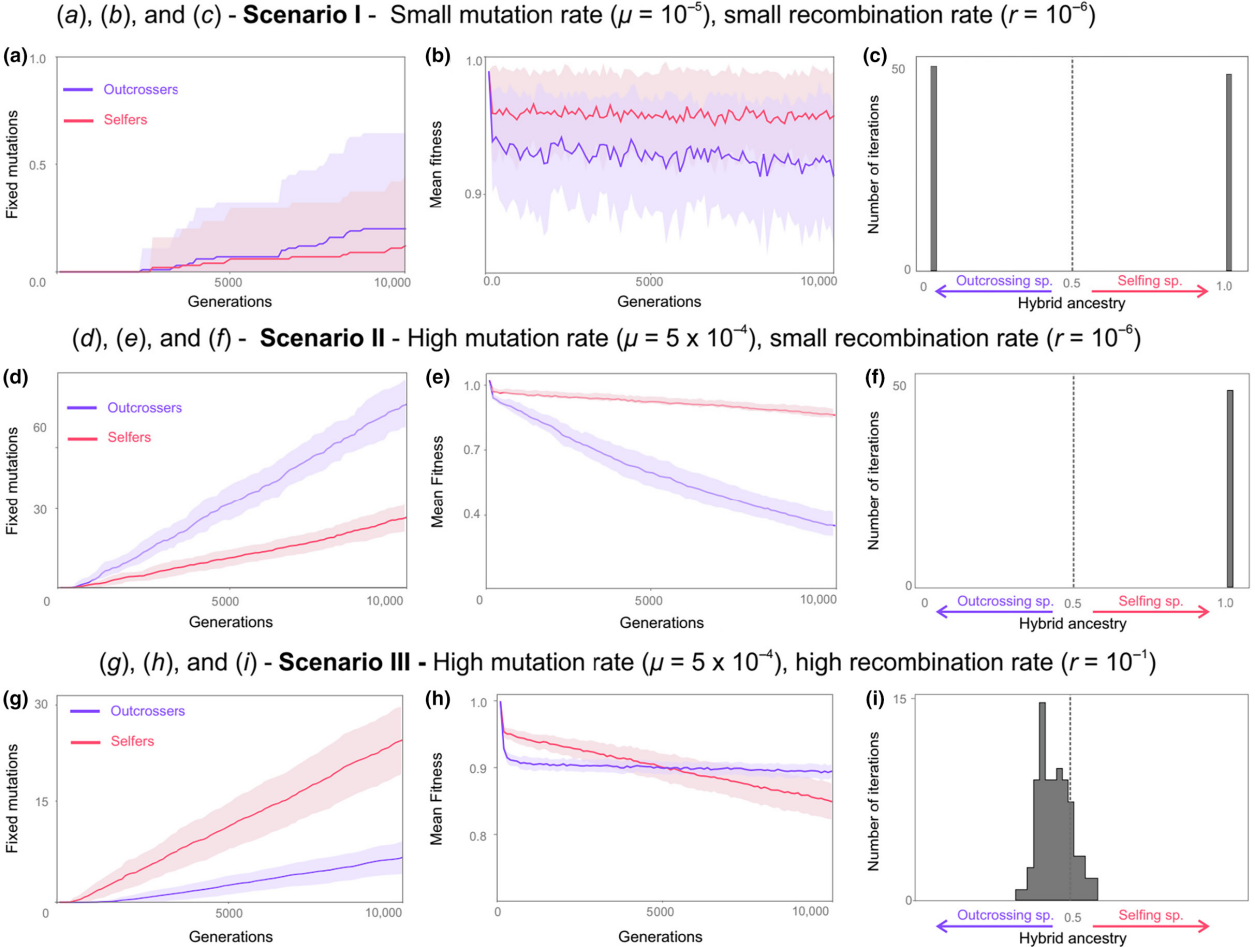
Consequences of the accumulation of mutation load in the parental populations on the genomic composition of hybrids. Here, an outcrossing (purple) and a self‐fertilizing (red) populations evolve for 10,000 generations. First column (a, d, g) shows the number of fixed deleterious mutations in their respective genomes across different mutation and recombination rates). Second column (b, e, h) shows the respective population mean fitnesses. Simulations are repeated 100 times: plain lines account for average values, while shaded areas display the standard deviation. After 10,000 generations, the parental populations hybridize, and we look at the genomic composition of the hybrid population after 20,000 additional generations (for this simulation the hybrid population reproduces by outcrossing). Column three (c, f, i) displays histograms of the hybrid ancestry (>0.5, selfing ancestry; <0.5 outcrossing ancestry). A gray vertical line indicates unbiased hybrid ancestry. Simulations show results for three different combinations of mutation and recombination rates: (a–c) Scenario I: Small mutation rate (*μ* = 10^−5^), small recombination rate (*r* = 10^−6^). (d–f) Scenario II: High mutation rate (*μ* = 5.10^−4^), small recombination rate (*r* = 10^−6^). (g–i) Scenario III: High mutation rate (*μ* = 5.10^−4^), high recombination rate (*r* = 10^−1^).

In the following, we look at the proportions of genes in an outcrossing hybrid population coming from each of the parental populations. In Figure 1, we illustrate the genomic composition of hybrid populations 20,000 generations after hybridization, considering a case where the parental outcrossing and self‐fertilizing populations have diverged during 10,000 generations prior to hybridization. We associate these patterns with the mutation load built up in the parental populations.

Figure 1 exemplifies three different scenarios. In the first scenario with small mutation and recombination rates (Figure 1, Scenario I), the mutation rate and time left for mutation accumulation are so small that mutation load barely builds up. Outcrossing and self‐fertilizing populations accumulate similar amounts of deleterious mutations. As a result, they do not significantly differ in population mean fitness. The outcrossing population has lower fitness due to higher frequencies of highly deleterious, strongly recessive mutations, but the difference is marginal. As a result, in the hybrid population around half of the time the genes of the outcrossing parents prevail, half the time the genes of the self‐fertilizing parents prevail. We see that there is no case of mixture of genes of the two populations. This is because the recombination rate is too low here. Eventually, one haplotype goes to fixation, and because the recombination rate is so small (*r* = 10^−6^) this haplotype is one chromosome of either one of the parental populations. The two populations have similar mean fitness; in fact, they both have many chromosomes without any deleterious mutations. One of these “optimal” chromosomes goes to fixation in the hybrid population, and there is an almost equal probability that the “optimal” haplotype that goes to fixation is from either one of the parental species.

The results of Scenario I strongly rely on the fact that deleterious mutations are rare: the genomic mutation rate is only 0.002 in these simulations, which is similar to some empirical findings (Keightley & Caballero, 1997; Zhu et al., 2014). This results in that many genomes are completely free of deleterious mutations or harbor very few of them, leading to insignificantly small selective interference in the selfers (a result already known, see Lande et al., 1994), and in general inexistent fitness differences between selfers and outcrossers. In the following scenarios, we consider alternative cases with genomic mutation rates increased to a value of 0.1 (which is closer to other reported empirical findings, see Charlesworth et al., 2004; Eyre‐Walker & Keightley, 1999; Shaw et al., 2002) by increasing the mutation rate per locus to 5.10^−4^.

In Scenario II (Figure 1, Scenario II), there is a significant accumulation of deleterious mutations. Because the recombination rate is still low (*r* = 10^−6^), both the outcrossing and the self‐fertilizing species suffer from selective interference. Heterozygosity in the outcrossing population helps deleterious mutations go to fixation; as a result, we see in this scenario that the outcrossing species accumulates more fixed deleterious mutations than the self‐fertilizing species (Figure 1d). This translates in the outcrossers having a much lower population mean fitness after 10,000 generations than the selfers (Figure 1e). In this case, we see that the genes from the self‐fertilizing parents always prevail in hybrids (Figure 1f). Again, because the recombination rate is small, the haplotype that goes to fixation is an entire chromosome of one of the parental populations; here always the self‐fertilizers since their genomes are of much better quality (Figure 1, Scenario II).

In Scenario III (Figure 1, Scenario III), high recombination rates mean that the outcrossing species suffers much less from selective interference than the self‐fertilizing species. Therefore, it accumulates less fixed deleterious mutations (Figure 1g), and have a somewhat better fitness after 10,000 generations than the self‐fertilizing population species (Figure 1h). In this case, we see that on average the genes from the outcrossing species prevail in the hybrid genomes (Figure 1i). However, this time we also see that hybrid genomes are a mixture of genes from the two parental populations (Figure 1i). This is because here high recombination rates (*r* = 10^−1^) in the hybrids mean that a mosaic haplotype goes to fixation. Because the genomes of the outcrossing species are in average of better quality, this mosaic tends to contain more genes from the outcrossing parents than genes from the self‐fertilizing parent.

### 3.3 Effects of hybrid mating system on hybrid genome composition

**FIGURE 2:**
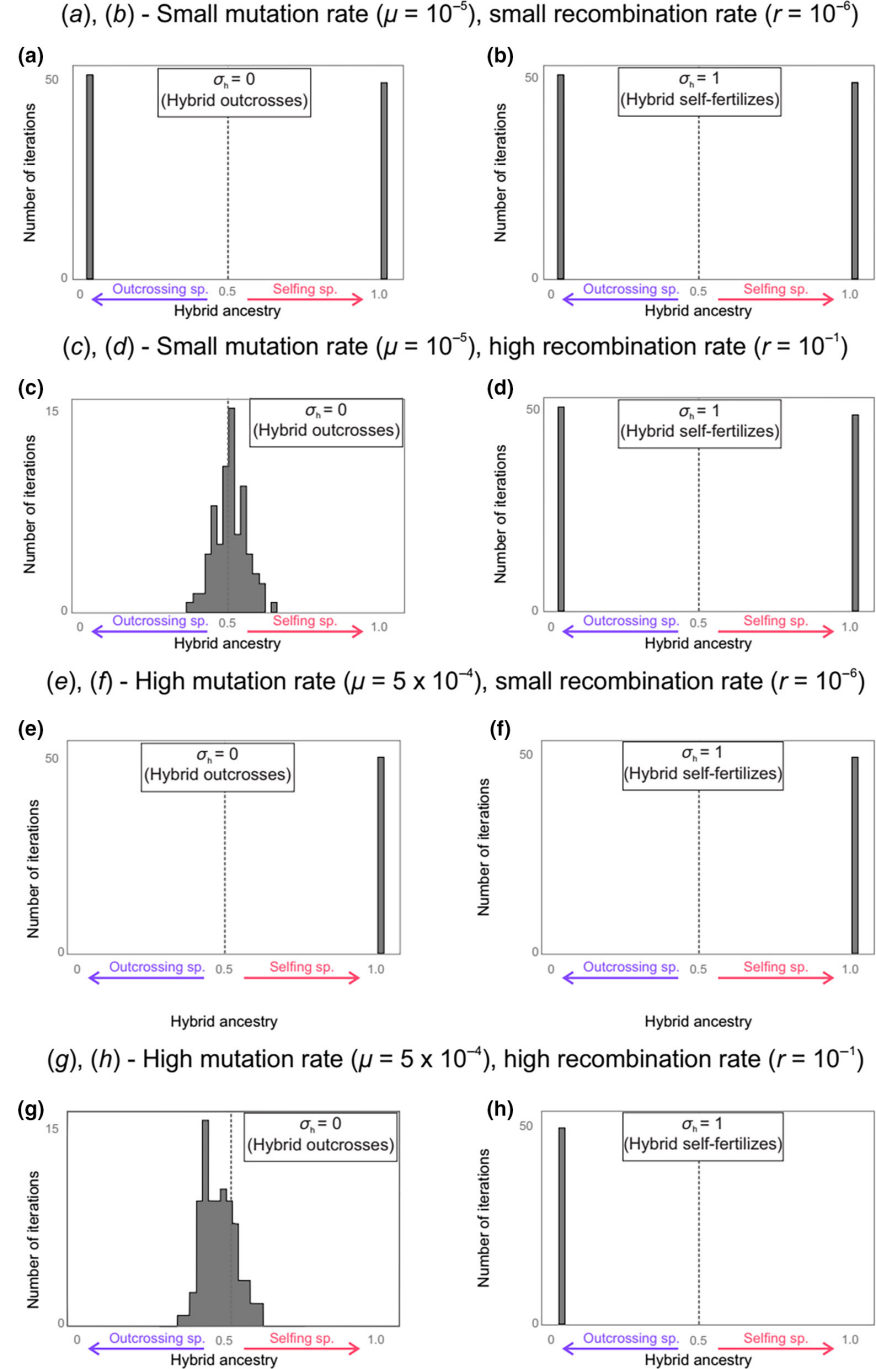
Hybrid genome composition depends on mutation rate (*μ*), recombination rate (*r*), and selfing rate of the hybrid (*σ*_h_). Results are displayed as proportion of the hybrid ancestry (>0.5, selfing ancestry; <0.5 outcrossing ancestry). A gray vertical line indicates unbiased hybrid ancestry. Hybrids have reproduced in isolation for 20,000 generations.

In Figure 1, the hybrid species always outcrosses to reproduce. To gain insights into how the mating system of the hybrid population impacts its genome composition, we show in Figure 2 histograms of hybrid genomes' ancestry for different values of *r*, *μ*, and *σ*_h_, the probability of hybrids to reproduce via self‐fertilization.

The mating system of the hybrid tends to not strongly modify the average ancestry of hybrid genes. When the genomes of the two parental populations are of similar quality (Figure 2a–d, *μ* = 1.10^−5^), hybrid genes have equal probability, on average, to come from the outcrossing or the self‐fertilizing parents. When the genomes of the self‐fertilizing parents tend to be of better quality (Figure 2e,f, *μ* = 5.10^−4^, *r* = 10^−6^), hybrid ancestry is entirely biased toward the self‐fertilizing parents, independently of whether the hybrid outcrosses (Figure 2e) or self‐fertilizes (Figure 2f). When the genomes of the outcrossing parents tend to be of better quality (Figure 2g,h, *μ* = 5.10^−4^, *r* = 10^−1^) is the only case where the reproductive mode of the hybrid appears to make significant differences. Whether the hybrids outcross or self‐fertilize, ancestry tends to be biased toward the outcrossing parent. However, ancestry is entirely biased toward the outcrossers when the hybrids self‐fertilize (Figure 2h), whereas it is only slightly biased toward the outcrossers when the hybrids outcross (Figure 2g).

The effect of the mating system of the hybrid thus seems secondary. It mostly appears when *r* = 10^−1^ (Figure 2b,d,g,h). In that case, there is a fundamental difference depending on how the hybrids reproduce. If the hybrids outcross, then their genomes are frequently recombined. As evoked earlier, this means that hybrid genomes become a mixture of the two parental populations, instead of being only composed of genes from one parental populations. When *μ* = 10^−5^ (Figure 2b,d), this mixture is unbiased since the two parental genomes are of similar quality. When *μ* = 5.10^−4^ (Figure 2g,h), the mixture is biased toward the outcrossing parents, as their genomes are of better quality. Overall, we see that the mating system of the hybrid does not change the direction of ancestry biases. It may only change its magnitude (strong or weak bias) and its modality (uniparental ancestry or mixed ancestry).

**FIGURE 3:**
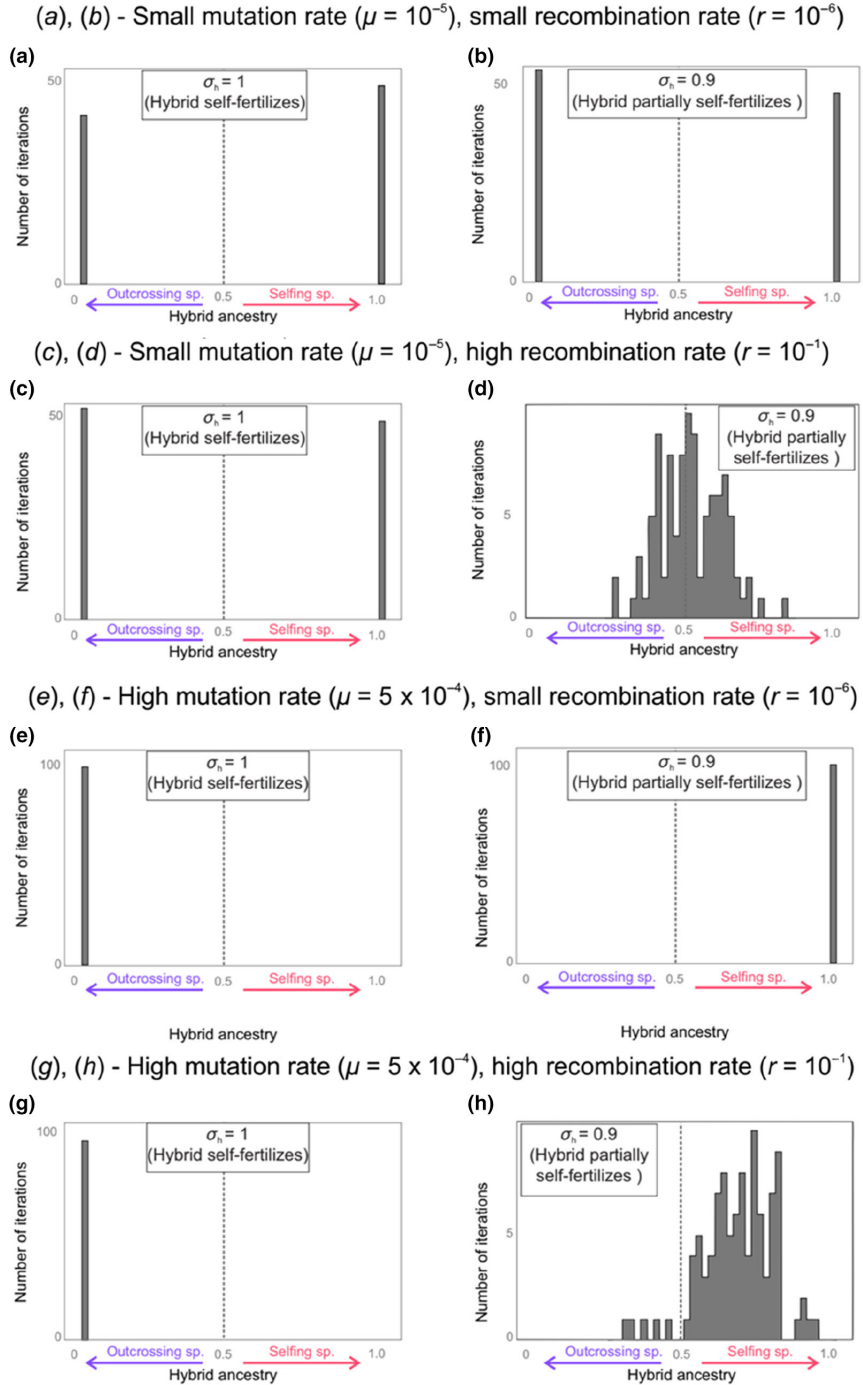
Hybrid genome composition also depends on the variation of selfing rate. Results are displayed as the proportion of the hybrid ancestry (>0.5, selfing ancestry; <0.5 outcrossing ancestry). Same values of r and μ are used as for Figure 2. We now compare *σ*_s_ = *σ*_h_ = 1 (a, c, e, g), with *σ*_s_ = *σ*_h_ = 0.9 (b, d, f, h). Histograms are shown for 100 iterations, with the x‐axis representing hybrid ancestry, 20,000 generations after hybridization. A gray vertical line indicates unbiased hybrid ancestry.

Figure 2 was drawn for complete self‐fertilization (*σ*_s_ = 1 and *σ*_h_ = 1). However, in nature, self‐fertilization rate is widely variable *g* (Jarne & Auld, 2006). To take that into account, we show in Figure 3 the effect of assuming self‐fertilization rates of only 90% (Figure 3).

We see that most of the time, adding low rates of outcrossing does not change the average ancestry in hybrids. Independently of whether the hybrids fully or mostly self‐fertilize, around 50% of hybrid genome comes from the self‐fertilizing parents when *μ* = 10^−5^ (Figure 3a–d), and only the self‐fertilizing parents' genes are transmitted to hybrids when *μ* = 5.10^−4^ and *r* = 10^−6^ (Figure 2e,f). However, patterns differ between *σ*_s_ = 1 and *σ*_s_ = 0.9 in the case where *μ* = 10^−5^ and *r* = 10^−1^ (Figure 3c,d). For these parameter values, only outcrossing parents' genes are inherited in hybrids when there is full self‐fertilization *σ*_s_ = *σ*_h_ = 1 (Figure 2e) while a majority of self‐fertilizing parents' genes are transmitted when *σ*_s_ = *σ*_h_ = 0.9 (Figure 2, h). We saw on Figure 1 Scenario III that for these numerical values of *μ* and *r*, outcrossing species' mean fitness just slightly overcome selfing species' mean fitness. When the self‐fertilization rate of the selfing species is decreased, it accumulates less deleterious mutations; here, this is just enough for the self‐fertilizing species' fitness to pass the outcrossing species's fitness, resulting in its genes being preferentially transmitted in hybrids. Overall, we see again that ancestry patterns will primarily depend on the accumulated mutation load in the parental species. As such, the mating system of the parental species matters: a 10% difference in selfing rate may be enough to switch the relative quality of the two parental populations, which in turn may result in large differences in hybrid ancestry.

A collateral effect of introducing low rates of outcrossing in self‐fertilizing hybrids is that it introduces small levels of effective recombination. This does not seem to have much of an effect when *r* = 10^−6^ (Figure 3a,b,e,f), as recombination rate is marginal. However, we see when *r* = 10^−1^ (Figure 3c,d,g,h) that this recombination allows for the hybrid genome to exhibit mixed ancestry, instead of being composed of genes from only one or the other parental species.

### 3.4 Effects of divergence time on hybrid genome composition

**FIGURE 4:**
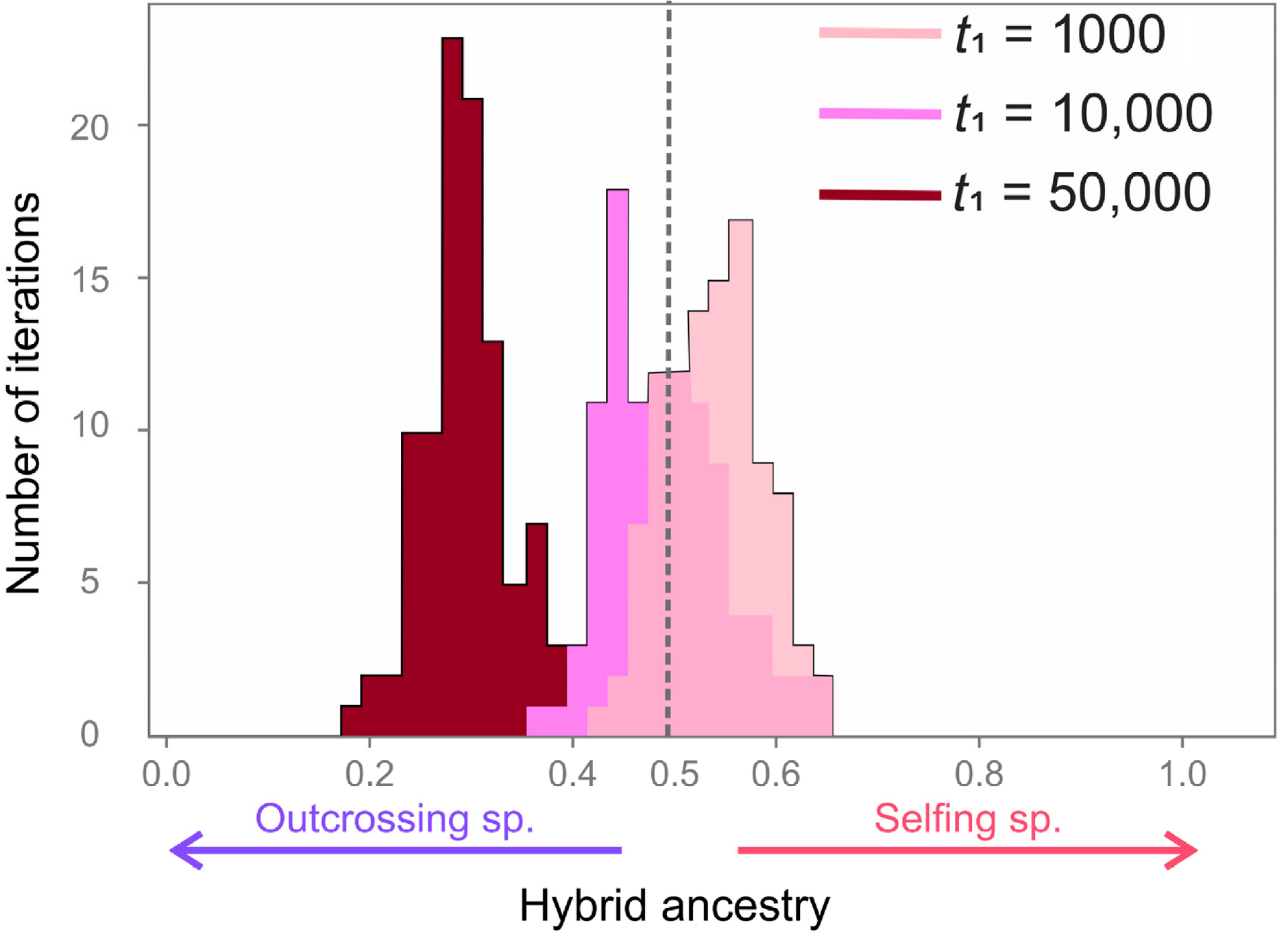
Hybrid ancestry depends on how much time passed before hybridization between parental selfers and outcrossers. Results are displayed as the proportion of the hybrid ancestry (>0.5, selfing ancestry; <0.5 outcrossing ancestry) for different number of generations of divergence between the parental populations prior to hybridization. We illustrate results for divergence times *t*_1_ = 1000 (lighter pink), *t*_1_ = 10,000 (pink) and *t*_1_ = 50,000 (darker red). Results are illustrated for 20,000 generations after hybridization.

When hybrids at least partially outcross and recombination is frequent, hybrids' genomes are composed of a mix of genes from the two parental populations (Figures 2c,g and 3d,h). In the case of a fully outcrossing hybrid, we illustrate in Figure 4 how the hybrid genome composition changes depending on the time of divergence prior to hybridization *t*_1_. As the populations have gone through longer divergence, the selfers´ genome is of worst quality compared with outcrossers’ genomes (see Figure 1 Scenario III). Consequently, and as illustrated in Figure 4, the part of hybrid genome descending from self‐fertilizing population ancestry tend to decrease with time prior to hybridization. Also, we see that the width of the distribution of ancestry decreases with increasing values of *t*_1_: as differences between outcrossers and selfers amplify, results of hybridization are more stable from one iteration to the next.

## 4 DISCUSSION

The transition from obligate outcrossing to predominantly selfing has happened multiple times in plants and animals (Jarne & Auld, 2006; Jarne & Chalesworth, 1993; The Tree of Sex Consortium, 2014). This transition comes with a combination of phenotypic and genomic changes collectively known as the “selfing syndrome” (Cutter, 2019; Shimizu & Tsuchimatsu, 2015). Among those changes, outcrossers and selfers are expected to differ in their mutation load. Theory predicts that higher levels of heterozygosity in outcrossing species allows to better hide mutations that are simultaneously highly deleterious and highly recessive from selection, resulting in these reaching higher frequencies in outcrossers when compared to selfers (Wang et al., 1999). In contrast, theory also predicts that higher levels of homozygosity in self‐fertilizing species result in less efficient recombination events, and thus stronger selective interference (Hartfield et al., 2017; Hartfield & Glémin, 2014, 2016; Wright et al., 2013). As a result, slightly deleterious, codominant alleles are more likely to escape being purged out by selection and increase in frequency by random genetic drift (Charlesworth et al., 1993). Our results generally agree with these predictions. As it turns out, there seem to be pros and cons for both outcrossing and self‐fertilization in terms of mutation load. An important question is, which mutation load is the highest and most influences the genetic content of outcrossing and selfing species?

Our results, in line with other recent theoretical results (Sianta et al., 2022) emphasize the importance of the recombination rate in the build‐up of the mutation load in outcrossers and selfers. We show that when recombination between adjacent loci is relatively high, outcrossers suffer little from selective interference. As a result, few deleterious mutations drift to fixation, fewer than in the self‐fertilizing species, which do suffer from selective interference. In this case, outcrossers tend to evolve better genomes than their self‐fertilizing counterparts (higher population mean fitness). Importantly, the prediction changes when we assume a low recombination rate between adjacent loci. In that case, outcrossers also suffer from selective interference since genetic associations are rarely reshuffled. In addition, higher heterozygosity in outcrossers means higher frequency of deleterious, recessive mutations, as their detrimental effects are partially hidden in heterozygotes. These higher frequencies lead to easier fixation for these deleterious mutations due to drift. As a result, we see that when recombination rates are low, outcrossers tend to accumulate more fixed deleterious mutations, which eventually translates into outcrossers evolving worse genomes (lower population mean fitness). This relates to the scarce empirical support that the theory of mutational meltdown in selfers has received (Escobar et al., 2010): we see that selfers are not necessarily predicted to accumulate more deleterious mutations than their outcrossing counterparts. Our work also emphasizes the role of the genomic mutation rate: there must be a sufficient influx of mutations for differences between selfers and outcrossers to appear over a reasonable timescale.

An interesting consequence of this result is that relative fitnesses may vary within genomes. Local rates of recombination are known to vary within genomes (Jensen‐Seaman et al., 2004; Kong et al., 2002; Nachman & Churchill, 1996). There is similar support for mutation rate variation within genomes (Hodgkinson & Eyre‐Walker, 2011; Nachman & Crowell, 2000; Wolfe et al., 1989). It is thus possible that hybrids of outcrossing and self‐fertilizing parents could end up with mosaic ancestry, with different parts of the genome having differently biased ancestry (Brandvain et al., 2014).

The accumulation of mutation load highly depends on genetic drift, and thus on the effective population size *N*_e_. On one hand, increasing population size limits the fixation of deleterious mutations. In simulations shown in Figure S3, with *μ* = 10^−5^ and *r* = 10^−6^, increasing population size from 500 to 5000 decreases the deleterious mutation fixation rate approximately by a factor of 10. On the other hand, there are particular demographic events that leads to *N*_e_ values much lower than the actual population size such as population bottlenecks or colonization events. Importantly, self‐fertilizing species seem to be prone to this kind of event as they can in principle more easily colonize new environments and found new populations (Grossenbacher et al., 2017; Noel et al., 2016; Theologidis et al., 2014). This may explain empirical patterns of relaxed purifying selection observed in some self‐fertilizing species (Burgarella et al., 2015; Slotte et al., 2010; Wang et al., 2021). Evidence for purifying selection in selfers is often mixed (Escobar et al., 2010; Glémin et al., 2006; Haudry et al., 2008). This mixed empirical evidence may be caused by the demographic history of the species as mentioned above but also due to other factors such as divergence time between selfers and outcrossers, biased gene conversion, stability of selfing as a reproductive strategy (Escobar et al., 2010), and negative linkage disequilibrium building up in selfers due to the inefficiency of recombination, which shelters deleterious mutations from selection (Abu Awad & Roze, 2018; Clo et al., 2020; Clo & Opedal, 2021; Lande & Porcher, 2015). Overall, it is likely that self‐fertilizing species suffer from greater mutation accumulation than what we predict here due to their demographic and evolutionary history.

How does mutation load in the parental species influence the ancestry of hybrids' genes? Previous studies have hypothesized that hybrid individuals' mating system should strongly impact the evolution of the genetic composition of hybrids and the levels of introgression to be expected in a hybrid zone. Pickup et al. (2019) predicts that in outcrossing hybrids, the many slightly deleterious, codominant mutations coming from the self‐fertilizing ancestry should be selected against and purged while the highly deleterious, recessive mutations coming from the outcrossing ancestry should be stay hidden in heterozygosity and be maintained. That is, the authors predict a purge of selfing ancestry in outcrossing hybrids. Similarly, they predict that in selfing hybrids, the highly deleterious, recessive mutations coming from the outcrossing ancestry should rapidly become homozygote and be selected against, while the many slightly deleterious, codominant mutations coming from the self‐fertilizing parent should keep on evading selection and be maintained. This time, the outcrossing ancestry is expected to be purged in selfing hybrids. Everything happens as if the mutation load was “adapted” to a certain reproductive mode and should be maintained in a hybrid that shares the same reproductive mode. This should result in limited introgression between outcrossing and self‐fertilizing species, in both directions.

Though appealing, this verbal argument does not appear to be verified in our simulations. Our results emphasize that what matters is merely which parental species has the strongest mutation load. The genome ancestry with the worst fitness will most likely be purged in hybrids, independently of the hybrid mating system itself.

Kim et al. (2018) produced a similar model of introgression between outcrosser and selfer parental populations. In their simulations, they obtained that genomes from outcrossing populations are of better quality and predict limited introgression of selfer into outcrosser genomes and greater introgression of outcrosser into selfer genomes. A few genomic parameters differ between our work and this paper. First, the authors use a gamma distribution for the selective coefficient of new deleterious mutations whose shape and scale parameters are such that overall selection is much weaker in their study. This may alter the mutation load balance by increasing genetic drift. However, it increases drift in both outcrossers and selfers, and simulations run with *s* = 0.01 (which provides a selective coefficient distribution much closer to the one in Kim et al. (2018)) show an acceleration of deleterious mutation accumulation similar in the two parental species (see Figure S4). Kim et al. (2018) also consider a genomic structure based on chromosome 1 of *Arabidopsis thaliana*. This notably means that they consider a greater number of loci under purifying selection, which is expected to increase the relative importance of selective interference and mutation accumulation. However, simulations with 1000 and 10,000 instead of 100 loci (Figure S5) again emphasize that mutation accumulation increases similarly in both selfers and outcrossers; in both cases, outcrossers still accumulate more deleterious mutations in our simulations.

Focusing on the case of *Arabidopsis thaliana*, Kim et al. (2018) acknowledge that they look at a scenario with relatively high recombination rates. As such, they may simply fall in a scenario where selective interference may play a stronger role in selfers (high recombination rates, see Figure 1 Scenario III). How their result is generalizable to other self‐fertilizing plants and animals remains to be assessed.

In this work, we used relatively simple and straightforward selective effects: fitness is multiplicative, only deleterious mutations are considered, and dominance of the mutations is a monotonous function of the strength of their detrimental effect. We do not consider cases of epistasis, beneficial mutations, compensatory or reverse mutations, or pleiotropic effects of mutations. We assume no difference in the environment nor in the population size of the two parental populations. This is a deliberate choice: providing a simple baseline model to focus on the effects of mating systems and mutation load on hybrid ancestry. All types of alteration of the model can potentially modify quantitatively but also qualitatively, the outcomes. What this model emphasizes is how the mutation load of the parental species is what primarily matters, and only secondarily the mating system of the hybrid population. How mutation load is influenced by the genetic architecture of the fitness is beyond the scope of the paper and is almost an infinite horizon of possible models. In that respect, the possibilities that SLiM offers to model genomes as close as possible to real genomes is a unique feature that could be exploited in the future to look at a variety of model organisms, as Kim et al. (2018) did for chromosome 1 of *Arabidopsis thaliana*.

Another important assumption of our model is that the hybrids reproduce with a fixed self‐fertilization rate *σ*_s_, independently of their genomic composition. In other words, we assume that none of the loci we model are involved in the determination of the self‐fertilization rate, or that self‐fertilization can be environmentally determined. An alternative would be to consider that the genomic composition of the hybrids directly influences the rate of self‐fertilization—this would mean that some of the loci modeled are involved in self‐fertilization rate determination. Such a case, though interesting, is beyond the scope of this paper: predictions are not trivial; as such, this model requires attentive examination.

Mutation load is not the only factor that may influence introgression patterns between self‐fertilizing and outcrossing species. Behavioral and physiological constraints are also major factors that are known to affect hybridization and introgression directions. In a hybrid zone between species with different mating systems, introgression is expected to happen more frequently from the selfing species to the outcrosser, than vice‐versa (Kim et al., 2018; Pickup et al., 2019). This expectation relies on the premise that selfing (especially prior selfing; Berbel‐Filho et al., 2021; Brys et al., 2016; Tian‐Bi et al., 2008) provides a very limited window of opportunity for outcrossing by conspecific, heterospecific, or potential male hybrids (Berbel‐Filho et al., 2021; Pickup et al., 2019). This pattern of higher levels of introgression from selfers into outcrossers has been commonly found in plant systems (Brandvain et al., 2014; Ruhsam et al., 2011, 2013). Our findings extend that expectation but from a different perspective. Our results indicate that under certain scenarios (here, lower recombination rates) self‐fertilizing species may exhibit lower mutation load relative to outcrossing species. Following what we uncovered here, this should be expected to bias hybrid ancestry toward the selfer parents that is to result in higher levels of introgression from selfers to outcrossers. However, under other scenarios (here, higher recombination rates), our model predicts the contrary: that hybrid ancestry would be biased toward the outcrossing, fitter parents.

We argue that our model provides insights into one of the factors, mutation load that may influence the direction of introgression in hybrids zones between selfers and outcrossers. Of course, many other factors also influence such introgression and should be taken into account when comparing introgression patterns with parental species' mutation load. For instance, the weak inbreeder/strong outbreeder hypothesis (WISO) (Brandvain & Haig, 2005) states that due to the enhanced opportunity for genomic conflict, outcrossers' gametes are more competitive than selfers' ones. Given equal fertilization opportunities, if hybrids mostly outcross, higher introgression is expected from outcrossers to selfers under the WISO premises. Another factor is selfing timing. Selfing can happen before (prior selfing), during (competitive), or after (delayed selfing) the opportunity for outcrossing (Lloyd, 1979). The parental selfing time may limit the window of opportunity from an outcrossing hybrid to the selfing parental species, making the major direction of hybridization more common from selfer to outcrosser, than vice‐versa (Berbel‐Filho et al., 2021; Brys et al., 2016). Finally, unilateral genetic incompatibilities may bias the direction of introgression (Turelli & Moyle, 2007) in both directions, regardless of the hybrids mating system and recombination rates. In our work, we focused solely on the effect of mating systems via mutation loads and have shown that parental species' loads are good predictors of the long‐term genomic composition of hybrid species. Altogether, the examples presented above represent interesting avenues of research whenever deviations from this prediction are found.

## AUTHOR CONTRIBUTIONS

**Fréderic Fyon:** Conceptualization (equal); formal analysis (equal); investigation (equal); methodology (lead); software (lead); visualization (lead); writing – original draft (equal); writing – review and editing (equal). **Waldir M. Berbel‐Filho:** Conceptualization (equal); formal analysis (equal); investigation (equal); methodology (supporting); writing – original draft (equal); writing – review and editing (equal).

## Supporting information


Appendix S1

## Data Availability

The manuscript did not make use of any data.

## References

[ece310538-bib-0001] Abu Awad, D. , & Billiard, S. (2017). The double edged sword: The demographic consequences of the evolution of self‐fertilization. Evolution, 71, 1178–1190. 28262926 10.1111/evo.13222

[ece310538-bib-0002] Abu Awad, D. , & Roze, D. (2018). Effects of partial selfing on the equilibrium genetic variance, mutation load, and inbreeding depression under stabilizing selection. Evolution, 72, 751–769. 29442366 10.1111/evo.13449

[ece310538-bib-0003] Agrawal, A. F. , & Whitlock, M. C. (2011). Inferences about the distribution of dominance drawn from yeast gene knockout data. Genetics, 187, 553–566. 21098719 10.1534/genetics.110.124560 PMC3030496

[ece310538-bib-0004] Arunkumar, R. , Ness, R. W. , Wright, S. I. , & Barrett, S. C. H. (2015). The evolution of selfing is accompanied by reduced efficacy of selection and purging of deleterious mutations. Genetics, 199, 817–829. 25552275 10.1534/genetics.114.172809 PMC4349074

[ece310538-bib-0005] Berbel‐Filho, W. M. , Tatarenkov, A. , Pacheco, G. , Espírito‐Santo, H. M. V. , Lira, M. G. , Garcia de Leaniz, C. , Avise, J. C. , Lima, S. M. Q. , Rodríguez‐López, C. M. , & Consuegra, S. (2021). Against the odds: Hybrid zones between mangrove killifish species with different mating systems. Genes, 12, 1486. 34680881 10.3390/genes12101486 PMC8535463

[ece310538-bib-0006] Brandvain, Y. , & Haig, D. (2005). Divergent mating systems and parental conflict as a barrier to hybridization in flowering plants. The American Naturalist, 166, 330–338. 10.1086/432036 16224688

[ece310538-bib-0007] Brandvain, Y. , Kenney, A. M. , Flagel, L. , Coop, G. , & Sweigart, A. L. (2014). Speciation and introgression between *Mimulus nasutus* and *Mimulus guttatus* . PLoS Genetics, 10, e1004410. 24967630 10.1371/journal.pgen.1004410 PMC4072524

[ece310538-bib-0008] Brys, R. , Van Cauwenberghe, J. , & Jacquemyn, H. (2016). The importance of autonomous selfing in preventing hybridization in three closely related plant species. Journal of Ecology, 104, 601–610.

[ece310538-bib-0009] Burgarella, C. , Gayral, P. , Ballenghien, M. , Bernard, A. , David, P. , Jarne, P. , Correa, A. , Hurtrez‐Boussès, S. , Escobar, J. , Galtier, N. , & Glémin, S. (2015). Molecular evolution of freshwater snails with contrasting mating systems. Molecular Biology and Evolution, 32, 2403–2416. 25980005 10.1093/molbev/msv121

[ece310538-bib-0010] Busch, J. W. , Bodbyl‐Roels, S. , Tusuubira, S. , & Kelly, J. K. (2022). Pollinator loss causes rapid adaptive evolution of selfing and dramatically reduces genome‐wide genetic variability. Evolution, 76, 2130–2144. 35852008 10.1111/evo.14572 PMC9543508

[ece310538-bib-0011] Charlesworth, B. , Borthwick, H. , Bartolomé, C. , & Pignatelli, P. (2004). Estimates of the genomic mutation rate for detrimental alleles in *Drosophila melanogaster* . Genetics, 167, 815–826. 15238530 10.1534/genetics.103.025262 PMC1470907

[ece310538-bib-0012] Charlesworth, D. , Morgan, M. T. , & Charlesworth, B. (1993). Mutation accumulation in finite outbreeding and inbreeding populations. Genetical Research, 61, 39–56.

[ece310538-bib-0013] Clo, J. , & Opedal, Ø. H. (2021). Genetics of quantitative traits with dominance under stabilizing and directional selection in partially selfing species. Evolution, 75, 1920–1935. 34219233 10.1111/evo.14304

[ece310538-bib-0014] Clo, J. , Ronfort, J. , & Abu Awad, D. (2020). Hidden genetic variance contributes to increase the short‐term adaptive potential of selfing populations. Journal of Evolutionary Biology, 33, 1203–1215. 32516463 10.1111/jeb.13660

[ece310538-bib-0015] Cutter, A. D. (2019). Reproductive transitions in plants and animals: Selfing syndrome, sexual selection and speciation. New Phytologist, 224, 1080–1094. 31336389 10.1111/nph.16075

[ece310538-bib-0016] Escobar, J. S. , Cenci, A. , Bolognini, J. , Haudry, A. , Laurent, S. , David, J. , & Glémin, S. (2010). An integrative test of the dead‐end hypothesis of selfing evolution in triticeae (Poaceae). Evolution, 64, 2855–2872. 20500214 10.1111/j.1558-5646.2010.01045.x

[ece310538-bib-0017] Eyre‐Walker, A. , & Keightley, P. (1999). High genomic deleterious mutation rates in hominids. Nature, 397, 344–347. 9950425 10.1038/16915

[ece310538-bib-0019] Gabriel, W. , Lynch, M. , & Bürger, R. (1993). Muller's ratchet and mutational meltdowns. Evolution, 47, 1744–1757. 28567994 10.1111/j.1558-5646.1993.tb01266.x

[ece310538-bib-0020] García‐Dorado, A. , & Caballero, A. (2000). On the average coefficient of dominance of deleterious spontaneous mutations. Genetics, 155, 1991–2001. 10924491 10.1093/genetics/155.4.1991 PMC1461187

[ece310538-bib-0021] Glémin, S. , Bazin, E. , & Charlesworth, D. (2006). Impact of mating systems on patterns of sequence polymorphism in flowering plants. Proceedings of the Royal Society B: Biological Sciences, 273, 3011–3019. 10.1098/rspb.2006.3657 PMC1639510 17015349

[ece310538-bib-0022] Grossenbacher, D. L. , Brandvain, Y. , Auld, J. R. , Burd, M. , Cheptou, P. O. , Conner, J. K. , Grant, A. G. , Hovick, S. M. , Pannell, J. R. , Pauw, A. , Petanidou, T. , Randle, A. M. , Rubio de Casas, R. , Vamosi, J. , Winn, A. , Igic, B. , Busch, J. W. , Kalisz, S. , & Goldberg, E. E. (2017). Self‐compatibility is over‐represented on islands. New Phytologist, 215, 469–478. 28382619 10.1111/nph.14534

[ece310538-bib-0023] Haller, B. C. , & Messer, P. W. (2019). SLiM 3: Forward genetic simulations beyond the Wright–fisher model. Molecular Biology and Evolution, 36, 632–637. 30517680 10.1093/molbev/msy228 PMC6389312

[ece310538-bib-0024] Hartfield, M. , Bataillon, T. , & Glémin, S. (2017). The evolutionary interplay between adaptation and self‐fertilization. Trends in Genetics, 33, 420–431. 28495267 10.1016/j.tig.2017.04.002 PMC5450926

[ece310538-bib-0025] Hartfield, M. , & Glémin, S. (2014). Hitchhiking of deleterious alleles and the cost of adaptation in partially selfing species. Genetics, 196, 281–293. 24240529 10.1534/genetics.113.158196 PMC3872191

[ece310538-bib-0026] Hartfield, M. , & Glémin, S. (2016). Limits to adaptation in partially selfing species. Genetics, 203, 959–974. 27098913 10.1534/genetics.116.188821 PMC4896205

[ece310538-bib-0027] Haudry, A. , Cenci, A. , Guilhaumon, C. , Paux, E. , Poirier, S. , Santoni, S. , David, J. , & Glémin, S. (2008). Mating system and recombination affect molecular evolution in four *Triticeae* species. Genetics Research, 90, 97–109. 18289404 10.1017/S0016672307009032

[ece310538-bib-0028] Hodgkinson, A. , & Eyre‐Walker, A. (2011). Variation in the mutation rate across mammalian genomes. Nature Review Genetics, 12, 756–766. 10.1038/nrg3098 21969038

[ece310538-bib-0029] Hu, X. S. (2015). Mating system as a barrier to gene flow. Evolution, 69, 1158–1177. 25873333 10.1111/evo.12660

[ece310538-bib-0031] Jarne, P. , & Auld, J. R. (2006). Animals mix it up too: The distribution of self‐fertilization among hermaphroditic animals. Evolution, 60, 1816–1824. 17089966 10.1554/06-246.1

[ece310538-bib-0032] Jarne, P. , & Chalesworth, D. (1993). The evolution of the selfing rate in functionally hermaphrodite plants and animals. Annual Review of Ecology, Evolution, and Systematics, 24, 441–466.

[ece310538-bib-0033] Jensen‐Seaman, M. I. , Furey, T. S. , Payseur, B. A. , Lu, Y. , Roskin, K. M. , Chen, C. F. , Thomas, M. A. , Haussler, D. , & Jacob, H. J. (2004). Comparative recombination rates in the rat, mouse, and human genomes. Genome Research, 14, 528–538. 15059993 10.1101/gr.1970304 PMC383296

[ece310538-bib-0034] Keightley, P. D. , & Caballero, A. (1997). Genomic mutation rates for lifetime reproductive output and lifespan in *Caenorhabditis elegans* . Proceeding of the Nationall Academy of Sciences, 94, 3823–3827. 10.1073/pnas.94.8.3823 PMC20525 9108062

[ece310538-bib-0035] Kim, B. Y. , Huber, C. D. , & Lohmueller, K. E. (2018). Deleterious variation shapes the genomic landscape of introgression. PLoS Genetics, 14, e1007741. 30346959 10.1371/journal.pgen.1007741 PMC6233928

[ece310538-bib-0036] Kong, A. , Gudbjartsson, D. F. , Sainz, J. , Jonsdottir, G. M. , Gudjonsson, S. A. , Richardsson, B. , Sigurdardottir, S. , Barnard, J. , Hallbeck, B. , Masson, G. , Shlien, A. , Palsson, S. T. , Frigge, M. L. , Thorgeirsson, T. E. , Gulcher, J. R. , & Stefansson, K. (2002). A high‐resolution recombination map of the human genome. Nature Genetics, 31, 241–247. 12053178 10.1038/ng917

[ece310538-bib-0037] Lande, R. , & Porcher, E. (2015). Maintenance of quantitative genetic variance under partial self‐fertilization, with implications for evolution of selfing. Genetics, 200, 891–906. 25969460 10.1534/genetics.115.176693 PMC4512550

[ece310538-bib-0038] Lande, R. , Schemske, D. W. , & Schultz, S. T. (1994). High inbreeding depression, selective interference among loci, and the threshold selfing rate for purging recessive lethal mutations. Evolution, 48, 965–978. 28564486 10.1111/j.1558-5646.1994.tb05286.x

[ece310538-bib-0039] Lloyd, D. G. (1979). Some reproductive factors affecting the selection of self‐fertilization in plants. The American Naturalist, 113, 67–79.

[ece310538-bib-0040] Lynch, M. , Bürger, R. , Butcher, D. , & Gabriel, W. (1993). The mutational meltdown in asexual populations. Journal of Heredity, 84, 339–344. 8409355 10.1093/oxfordjournals.jhered.a111354

[ece310538-bib-0041] Lynch, M. , Conery, J. , & Bürger, R. (1995). Mutational meltdowns in sexual populations. Evolution, 49, 1067–1080. 28568521 10.1111/j.1558-5646.1995.tb04434.x

[ece310538-bib-0042] Manna, F. , Martin, G. , & Lenormand, T. (2011). Fitness landscapes: An alternative theory for the dominance of mutation. Genetics, 189, 923–937. 21890744 10.1534/genetics.111.132944 PMC3213354

[ece310538-bib-0043] Martin, N. H. , & Willis, J. H. (2007). Divergence associated with mating system causes nearly complete reproductive isolation between sympatric Mimulus species. Evolution, 61, 68–82. 17300428 10.1111/j.1558-5646.2007.00006.x

[ece310538-bib-0044] Nachman, M. W. , & Churchill, G. A. (1996). Heterogeneity in rates of recombination across the mouse genome. Genetics, 142, 537–548. 8852851 10.1093/genetics/142.2.537 PMC1206986

[ece310538-bib-0045] Nachman, M. W. , & Crowell, S. L. (2000). Estimate of the mutation rate per nucleotide in humans. Genetics, 156, 297–304. 10978293 10.1093/genetics/156.1.297 PMC1461236

[ece310538-bib-0046] Noël, E. , Chemtob, Y. , Janicke, T. , Sarda, V. , Pélissié, B. , Jarne, P. , & David, P. (2016). Reduced mate availability leads to evolution of self‐fertilization and purging of inbreeding depression in a hermaphrodite. Evolution, 70, 625–640. 26899922 10.1111/evo.12886

[ece310538-bib-0047] Ostevik, K. L. , Rifkin, J. L. , Xia, H. , & Rausher, M. D. (2021). Morning glory species co‐occurrence is associated with asymmetrically decreased and cascading reproductive isolation. Evolution Letters, 5, 75–85. 33552537 10.1002/evl3.205 PMC7857285

[ece310538-bib-0048] Phadnis, N. , & Fry, J. D. (2005). Widespread correlations between dominance and homozygous effects of mutations: Implications for theories of dominance. Genetics, 171, 385–392. 15972465 10.1534/genetics.104.039016 PMC1456530

[ece310538-bib-0049] Pickup, M. , Brandvain, Y. , Fraïsse, C. , Yakimowski, S. , Barton, N. H. , Dixit, T. , Lexer, C. , Cereghetti, E. , & Field, D. L. (2019). Mating system variation in hybrid zones: Facilitation, barriers and asymmetries to gene flow. New Phytologist, 224, 1035–1047. 31505037 10.1111/nph.16180 PMC6856794

[ece310538-bib-0052] Ruhsam, M. , Hollingsworth, P. , & Ennos, R. (2011). Early evolution in a hybrid swarm between outcrossing and selfing lineages in Geum. Heredity, 107, 246–255. 21448227 10.1038/hdy.2011.9 PMC3183954

[ece310538-bib-0053] Ruhsam, M. , Hollingsworth, P. M. , & Ennos, R. A. (2013). Patterns of mating, generation of diversity, and fitness of offspring in a Geum hybrid swarm. Evolution, 67, 2728–2740. 24033179 10.1111/evo.12147

[ece310538-bib-0054] Shaw, F. H. , Geyer, C. J. , & Shaw, R. G. (2002). A comprehensive model of mutations affecting fitness and inferences for *Arabidopsis thaliana* . Evolution, 56, 453–463. 11989677 10.1111/j.0014-3820.2002.tb01358.x

[ece310538-bib-0055] Shimizu, K. K. , & Tsuchimatsu, T. (2015). Evolution of selfing: Recurrent patterns in molecular adaptation. Annual Review of Ecology, Evolution, and Systematics, 46, 593–622.

[ece310538-bib-0056] Sianta, S. A. , Peischl, S. , Moeller, D. A. , & Brandvain, Y. (2022). The efficacy of selection may increase or decrease with selfing depending upon the recombination environment. Evolution, 77, 394–408. 10.1093/evolut/qpac013 36622723

[ece310538-bib-0057] Simmons, M. J. , & Crow, J. F. (1977). Mutations affecting fitness in *drosophila* populations. Annual Review of Genetics, 11, 49–78. 10.1146/annurev.ge.11.120177.000405 413473

[ece310538-bib-0058] Slotte, T. , Foxe, J. P. , Hazzouri, K. M. , & Wright, S. I. (2010). Genome‐wide evidence for efficient positive and purifying selection in *Capsella grandiflora*, a plant species with a large effective population size. Molecular Biology and Evolution, 27, 1813–1821. 20194429 10.1093/molbev/msq062

[ece310538-bib-0060] The Tree of Sex Consortium . (2014). Tree of sex: A database of sexual systems. Scientific Data, 1, 140015. 25977773 10.1038/sdata.2014.15 PMC4322564

[ece310538-bib-0061] Theologidis, I. , Chelo, I. M. , Goy, C. , & Teotónio, H. (2014). Reproductive assurance drives transitions to self‐fertilization in experimental *Caenorhabditis elegans* . BMC Biology, 12, 1–21. 25369737 10.1186/s12915-014-0093-1 PMC4234830

[ece310538-bib-0062] Tian‐Bi, Y.‐N. T. , N'Goran, E. K. , N'Guetta, S. P. , Matthys, B. , Sangare, A. , & Jarne, P. (2008). Prior selfing and the selfing syndrome in animals: An experimental approach in the freshwater snail *Biomphalaria pfeifferi* . Genetics Research, 90, 61–72. 18289401 10.1017/S0016672307008919

[ece310538-bib-0063] Turelli, M. , & Moyle, L. C. (2007). Asymmetric postmating isolation: Darwin's corollary to Haldane's rule. Genetics, 176, 1059–1088. 17435235 10.1534/genetics.106.065979 PMC1894575

[ece310538-bib-0064] Wang, J. , Hill, W. G. , Charlesworth, D. , & Charlesworth, B. (1999). Dynamics of inbreeding depression due to deleterious mutations in small populations: Mutation parameters and inbreeding rate. Genetical Research, 74, 165–178. 10584559 10.1017/s0016672399003900

[ece310538-bib-0065] Wang, X.‐J. , Barrett, S. C. H. , Zhong, L. , Wu, Z.‐K. , Li, D.‐Z. , Wang, H. , & Zhou, W. (2021). The genomic selfing syndrome accompanies the evolutionary breakdown of heterostyly. Molecular Biology and Evolution, 38, 168–180. 32761213 10.1093/molbev/msaa199 PMC7782863

[ece310538-bib-0066] Whitehead, M. R. , Lanfear, R. , Mitchell, R. J. , & Karron, J. D. (2018). Plant mating systems often vary widely among populations. Frontiers in Ecology and Evolution, 6, 38.

[ece310538-bib-0067] Willi, Y. (2013). Mutational meltdown in selfing *Arabidopsis lyrata* . Evolution, 67, 806–815. 23461329 10.1111/j.1558-5646.2012.01818.x

[ece310538-bib-0068] Wolfe, K. H. , Sharp, P. M. , & Li, W. H. (1989). Mutation rates differ among regions of the mammalian genome. Nature, 337, 283–285. 2911369 10.1038/337283a0

[ece310538-bib-0069] Wright, S. I. , Kalisz, S. , & Slotte, T. (2013). Evolutionary consequences of self‐fertilization in plants. Proceedings of the Royal Society B: Biological Sciences, 280, 20130133. 10.1098/rspb.2013.0133 PMC3652455 23595268

[ece310538-bib-0070] Zhu, Y. O. , Siegal, M. L. , Hall, D. W. , & Petrov, D. A. (2014). Precise estimates of mutation rate and spectrum in yeast. Proceedings of the National Academy of Sciences, 111, E2310–E2318. 10.1073/pnas.1323011111 PMC4050626 24847077

